# Spot the match – wildlife photo-identification using information theory

**DOI:** 10.1186/1742-9994-4-2

**Published:** 2007-01-16

**Authors:** Conrad W Speed, Mark G Meekan, Corey JA Bradshaw

**Affiliations:** 1 School for Environmental Research, Institute of Advanced Studies, Charles Darwin University, Darwin, Northern Territory 0909, Australia; 2 Australian Institute of Marine Science, P.O. Box 40197, Casuarina MC, Northern Territory 0811, Australia

## Abstract

**Background:**

Effective approaches for the management and conservation of wildlife populations require a sound knowledge of population demographics, and this is often only possible through mark-recapture studies. We applied an automated spot-recognition program (I^3^S) for matching natural markings of wildlife that is based on a novel information-theoretic approach to incorporate matching uncertainty. Using a photo-identification database of whale sharks (*Rhincodon typus*) as an example case, the information criterion (IC) algorithm we developed resulted in a parsimonious ranking of potential matches of individuals in an image library. Automated matches were compared to manual-matching results to test the performance of the software and algorithm.

**Results:**

Validation of matched and non-matched images provided a threshold IC weight (approximately 0.2) below which match certainty was not assured. Most images tested were assigned correctly; however, scores for the by-eye comparison were lower than expected, possibly due to the low sample size. The effect of increasing horizontal angle of sharks in images reduced matching likelihood considerably. There was a negative linear relationship between the number of matching spot pairs and matching score, but this relationship disappeared when using the IC algorithm.

**Conclusion:**

The software and use of easily applied information-theoretic scores of match parsimony provide a reliable and freely available method for individual identification of wildlife, with wide applications and the potential to improve mark-recapture studies without resorting to invasive marking techniques.

## Background

Effective approaches for the management and conservation of wildlife populations require a sound knowledge of population demographics [1]. For many species, such information is provided by studies that recognize individual animals so that their fate can be followed through time, thus allowing for the estimation of demographic rates like survival [2]. Individual recognition may be achieved either by applying an artificial mark to an animal or by using an animal's natural markings [3]. The former technique is pervasive in ecological studies addressing questions from the purely theoretical [e.g., [4]] to the highly applied [5], and it has been used on both marine and terrestrial species of vastly different sizes [e.g., [6,7]].

Applying artificial marks to wildlife can, however, alter natural behaviour and reduce individual performance [e.g., [8]]. The marking process itself may be disruptive [9] due to the necessity of handling and restraining for mark application [10]. The loss of marks over time [11] and the non-reporting of retrieved marks [12] can also compromise the estimation of demographic parameters. Additionally, there are often a host of ethical and welfare issues that can arise from the application of permanent or temporary marks [13,14].

To address some of these problems, the identification of individual animals from their natural markings has become a major tool for the study of some animal populations [15], and has been applied to an equally wide range of animals from badgers [16] to whales [17,18]. One of the more popular techniques of recording the natural markings of an animal is photo-identification as this allows storage of photos in a library for subsequent cross-matching and generation of capture-history matrices [17,19]. These libraries can be examined manually to develop a suite of individual matches [19]; however, as the number of photos in a library increases beyond a person's capacity to process the suite of candidate matches manually, the development of faster, automated techniques to compare new photographs to those previously obtained is required [20,21]. Several automated matching algorithms have been trialled with some success [e.g., [20,22-26]], but these are generally highly technical, specialized and target a particular taxon or unique morphological feature of the species in question (e.g., dorsal fin shape and markings in cetaceans). Furthermore, uncertainty in the matching algorithms themselves have never been contextualized within a multi-model inferential framework [27], and so subjective manual matching is still required to assess reliability [28].

An example taxon that lends itself well to the development and application of a generalist algorithm for photo matching is the world's largest fish – the whale shark (*Rhincodon typus*). This species has been the recent subject of several photo-identification studies [e.g., [19,20,29]], some of which have already provided valuable information on population size, structure [19] and demography [29] under the supported assertion that the spot and stripe patterns of animals are individually unique and temporally stable [19]. The initial assessment of the demography of one population (Ningaloo Reef, Western Australia) [19] has been complicated by the addition of many hundreds of photographs taken during analogous research programmes in other parts of Australia, Belize, USA, Philippines and Mexico [30], and elsewhere (Djibouti, Seychelles and Mozambique). Consequently, the number of photographs available has exceeded the number that can be reliably matched by eye, thereby necessitating an automated system of matching. One such system has been developed from an algorithm originally designed for stellar pattern recognition, and is currently being employed by the ECOCEAN whale shark database [20]. This system has great potential; however, the procedure for entering and matching patterns is complex, and neither the algorithm nor results are publicly available. Therefore, a simple, yet reliable algorithm accessible to the public is needed to incorporate effectively a large number of photographs from a wide range of researchers, tourist operators and private organizations. Such a software package has recently been developed and is known as Interactive Individual Identification System (I^3^S) [31,32].

**Figure 1:**
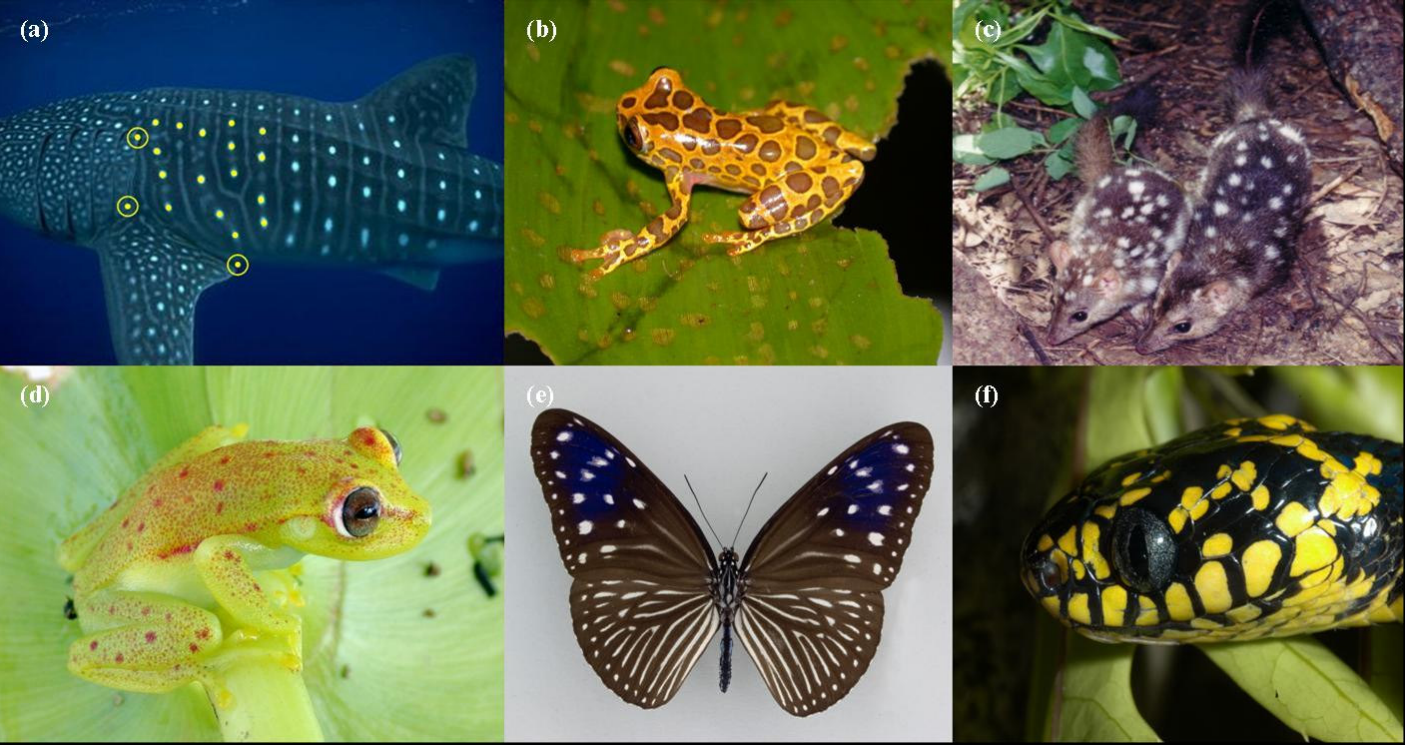
**Example species with sufficient spot patterning that could be useful for automated photo-identification**. Shown are (a) whale shark (*Rhincodon typus *– Photo ^© ^G. Taylor) indicating the reference area defined as the area encompassed by the reference points (yellow circles); (b) spotted tree frog (*Hyla leucophyllata *– Photo ^© ^D. Bickford); (c) northern quoll, (*Dasyurus hallucatus *– Photo ^© ^J. Kirwan); (d) Amazon spotted frog (*Hyla punctata *– Photo ^© ^D. Bickford); (e) striped blue crow (*Euploea mulciber *– Photo ^© ^D. Lohman); and (f) mangrove snake (*Boiga dendrophilia *– Photo ^© ^D. Bickford).

**Figure 2:**
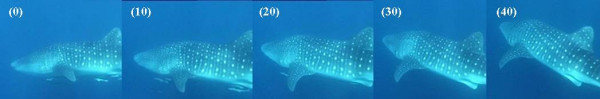
**An individual whale shark at varying angles of yaw (A: 0°, B: 10°, C: 20°, D: 30°, E: 40°)**. Sequences such as this were used to assess the effect of horizontal angle on the I^3^S matching process.

Our aim in this paper is to assess the reliability of this simple, freely available software package that recognizes spot patterns for use in photo-identification studies of wildlife. Although we focus on whale sharks as an example system, the application of the computer package and the information-theoretic matching algorithms we develop can be applied to any marine or terrestrial species demonstrating some form of stable spot patterning (e.g., sharks, frogs, lizards, mammals, butterflies, birds, etc. – Fig. 1). We assess the reliability of this package by comparing known matches made by eye. We also determine the effect of variation in the horizontal angle of subjects (Fig. 2) in matching reliability, as well as how the number of spot pairs in matched images affects matching performance. All matching results are developed within a fully information-theoretic framework that incorporates all of the uncertainty associated with the matching algorithm, thus aiding users in providing reliability assessments to their matches and the resulting capture histories and demographic estimates. As such, we provide a novel and parsimonious method for assessing the reliability of pattern matching applicable to a wide range of naturally identifiable wildlife species.

## Results

### I^3^S (Interactive Individual Identification Software) matching validation

**Figure 3:**
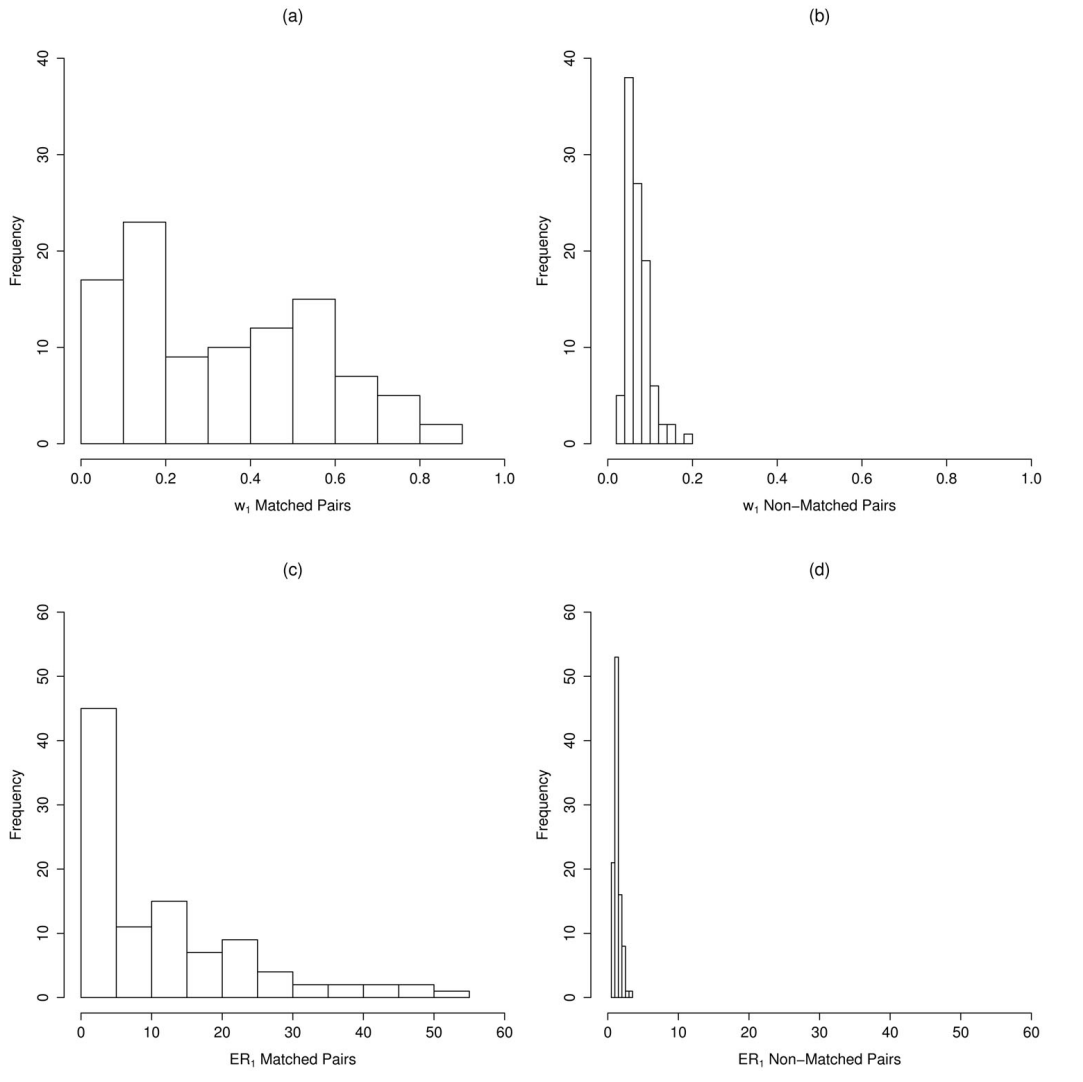
**I^3^S matching validation IC weights (*w*_1_)**. Distribution of IC weights for known matched (a) and non-matched pairs (b), and I^3^S matching validation evidence ratios (*ER*_1_) for known matched (c) and non-matched pairs (d) are shown.

The Information Criterion weights (*w*) for the most parsimonious matches (*w*_1_) for the 50 matched pairs (100 images) were broadly distributed between 0.05 and 0.85, while *w*_1 _for the 50 non-matched pairs (100 images) were highly right-skewed (Fig. 3a,b). All *w*_1 _for non-matched pairs were <0.18. The median *w*_1 _for matched pairs was 0.32 (± 0.05), which was much greater than the median for non-matched pairs (0.06 ± 0.01). Evidence ratios for the best-matched relative to the next-highest matched images (*ER*_1_) for known matched pairs were also highly right-skewed and ranged from 0.73 to 51.92, with a median of 7.36 (± 2.45) (Fig. 3c). *ER*_1 _for non-matched pairs were all <3.5 (median = 1.21 ± 0.09) (Fig. 3d). Evidence ratios for the second best-matched relative to the next-highest matched images (*ER*_2_) for known matched pairs ranged from 0.73 to 114.18, with a median of 7.57 (± 3.82). *ER*_2 _for non-matched pairs were also all <3.5 (median = 1.42 ± 0.12).

**Figure 4:**
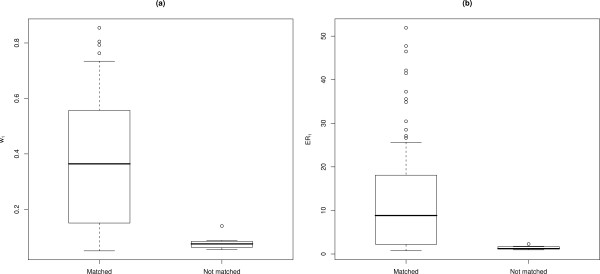
**Matching validation results**. Box-and-whisker plots of (a) IC weights (*w*_1_) for known matched pairs showing images matched and not matched with I^3^S; (b) evidence ratios (*ER*_1_) for known matched pairs showing images matched and not matched using I^3^S. Central tendency (black horizontal line) indicates the median, and whiskers extend to 0.5 of the inter-quartile range.

Overall, 93 images out of the 50 known-matched pairs were matched correctly using I^3^S. *w*_1 _for the correctly assigned matches ranged from 0.05 to 0.85 (median = 0.36 ± 0.05), and their *ER*_1 _ranged from 0.73 to 51.92 (median = 8.82 ± 2.56) (Fig. 4a,b). Known-matched photographs that I^3^S failed to match (7 images) had *w*_1 _that ranged from 0.05 to 0.14 (median = 0.07 ± 0.02), with their *ER*_1 _ranging from 0.95 to 2.28 (median = 1.23 ± 0.36).

### Assessing 'by-eye' matches using I^3^S

Of the 33 individuals re-sighted between years in the database used by Meekan et al. [19], 10 individuals could not be matched with I^3^S because their images were not amenable to I^3^S fingerprinting (absence of reference points) or their match was not present in the database. This was because the Meekan et al. [19] study also used images from a separate database and included scar-identified individuals that were not available for photographic matching using I^3^S. Thus, we could only re-assess 23 of these by-eye matches that included 13 LS matches and 16 RS matches (58 images total).

**Figure 5:**
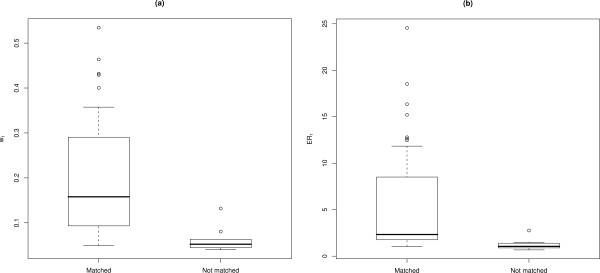
**Automated versus by-eye matching results**. Box-and-whisker plots of (a) IC weights (*w*_1_) for by-eye matched images that were matched and not matched using I^3^S; (b) Evidence ratios (*ER*_1_) for by-eye matched images that were matched and not matched using I^3^S. Central tendency (black horizontal line) indicates the median, and whiskers extend to 0.5 of the inter-quartile range.

Forty-eight of the 58 images (83%) from the 23 individuals were matched correctly using I^3^S. *w*_1 _for the correctly assigned by-eye matches ranged from 0.05 to 0.53 (median = 0.16 ± 0.04) (Fig. 5a), and their *ER*_1 _were between 1.04 and 24.57 (median = 2.33 ± 1.58) (Fig. 5b). Incorrectly assigned by-eye matches had *w*_1 _ranging from 0.04 to 0.13 (median = 0.06 ± 0.01) and their *ER*_1 _ranged from 0.67 to 2.76 (median = 1.04 ± 0.37). I^3^S also identified two images that were false positives (i.e., sharks that were incorrectly matched with other photographs) in the by-eye matching process. Neither of these images was matched with other known images of the identified sharks.

### Horizontal angle

**Figure 6:**
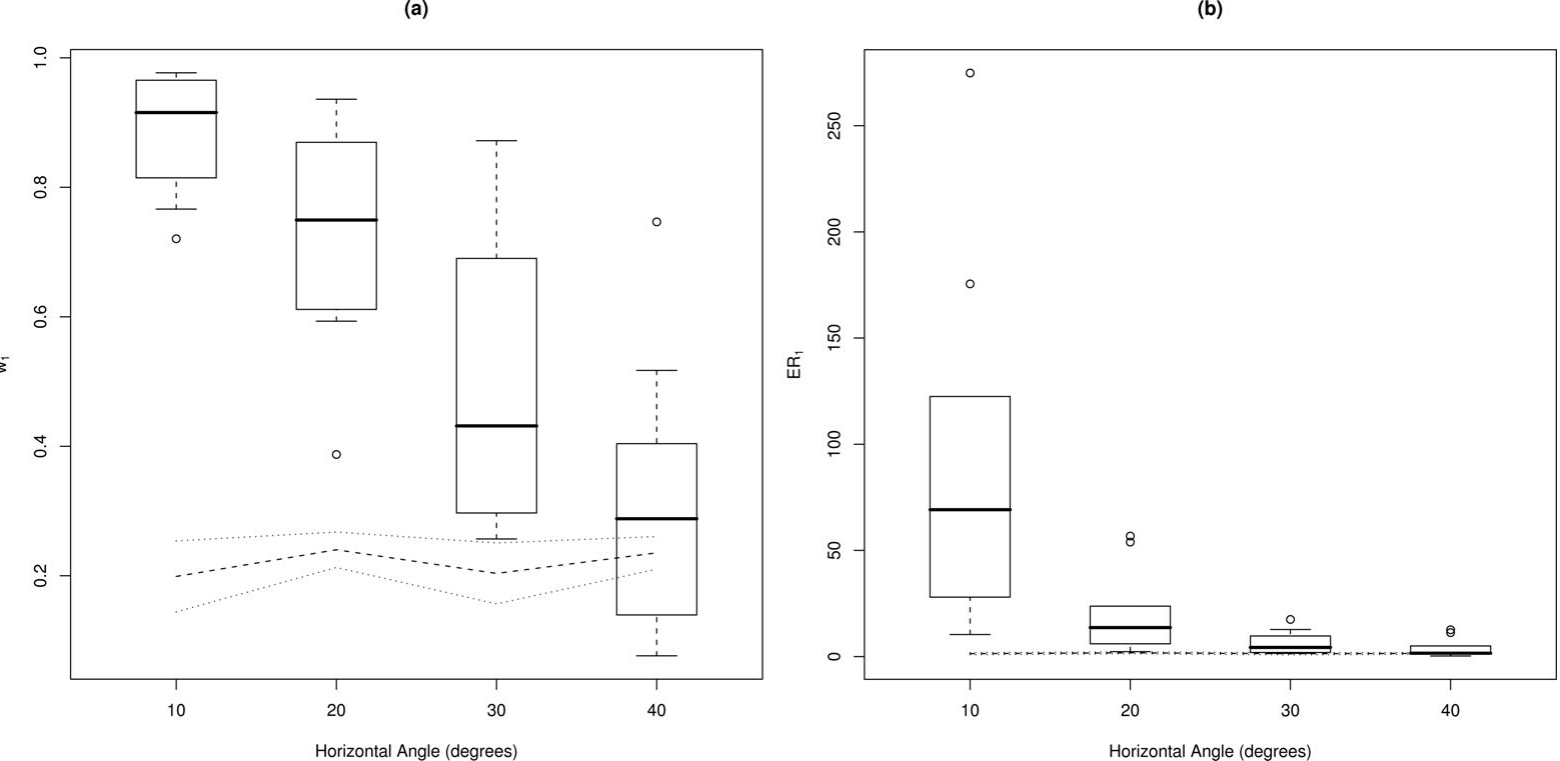
**Effect of angles of yaw**. Box-and-whisker plots of (a) IC weights (*w*_1_) for horizontal angle categories, where images at 0° were matched against images skewed by 10°, 20°, 30° and 40°. Dotted lines show results for non-matching pairs; (b) evidence ratios (*ER*_1_) for horizontal angle categories, where images at 0° were matched against images skewed by 10°, 20°, 30° and 40°. Central tendency (black horizontal line) indicates the median, and whiskers extend to 0.5 of the inter-quartile range.

Mean *w*_1 _decreased linearly as the horizontal angle of subjects within images increased (Fig. 6a). Median *w*_1 _ranged between 0.92 (± 0.06) for angles of 10°, to 0.29 (± 0.13) for angles of 40°. The images of subjects at 30° had *w*_1 _approaching those of non-matching pairs, and the distribution of *w*_1 _for images of subjects at 40° overlapped the distribution of *w*_1 _for non-matching pairs (Fig. 6a).

There was an exponential decline of median *ER*_1 _with increasing angle (Fig. 6b). Median *ER*_1 _ranged from 69.16 (± 52.24) for images of subjects at 10°, to 1.56 (± 2.81) for images of subjects at 40°. The distribution of *ER*_1 _for images of subjects at 30° approached that for non-matching pairs, and the distribution of *ER*_1 _for images of subjects at 40° overlapped the *ER*_1 _distribution for non-matching pairs.

### Number of spot pairs

**Figure 7:**
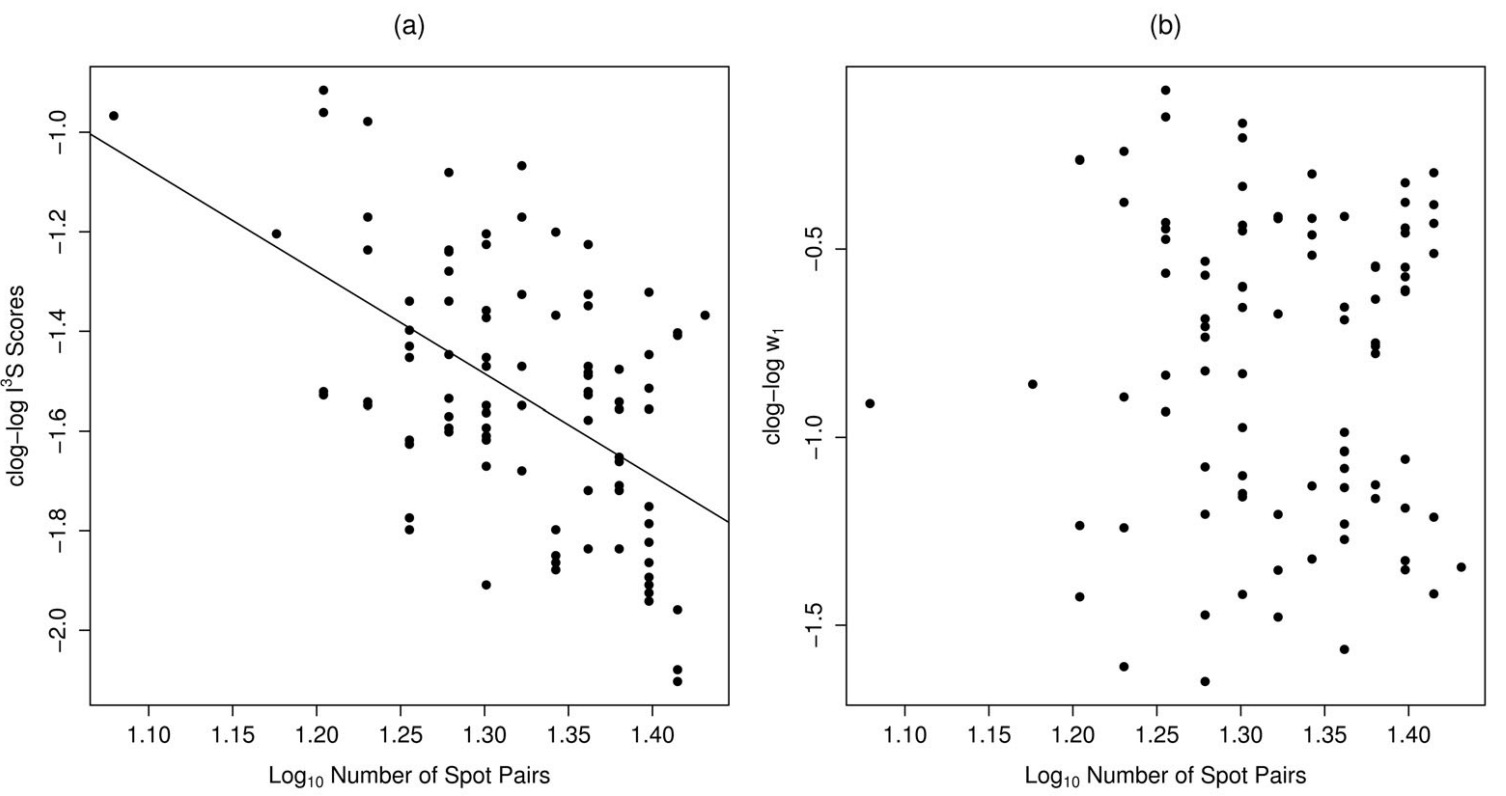
**Effects of spot-pair number**. (a) Relationship between complementary log-log-transformed (clog-log) I^3^S scores and log_10_-transformed number of spot pairs. The fitted line illustrates the correlation observed using a linear regression; (b) Comparison of clog-log-transformed *w*_1 _with log_10_-transformed number of spot pairs.

There was evidence for a negative relationship between the transformed I^3^S scores and spot pairs (*ER *= 9.94 × 10^5^, adjusted R^2 ^= 0.26; Fig. 7a), but no evidence for a relationship between *w*_1 _and the number of spot pairs (*ER *< 1; Fig. 7b).

## Discussion

Consistent, non-intrusive and ethically acceptable methods of mark-recapture are essential for estimating reliable demographic rates for wildlife populations, particularly for threatened species [29,33]. Photo-identification has become a widely accepted method of mark-recapture that has been empirically tested over a broad range of species [e.g., [16,17,34]]. Despite the advantages of this technique, there is the potential for large photographic databases to compromise the reliability of matches made by eye, which can subsequently jeopardize reliable estimates of population demographics. This problem has been largely overcome for several species by computer-aided image-matching algorithms that match various unique features of individuals [20,28,35-37]. However, most of these programs have limited applications, may be complex to operate, or are not freely available.

Software inaccessibility and the corresponding isolation of potentially useful photographic datasets will likely compromise parameter estimation and lead to higher uncertainty for calculated vital rates. For example, centralized photographic catalogues are common in the field of cetacean research, with new photographs from observers being compared to those previously obtained and the results sent to collaborators worldwide [38]. This type of data sharing for large, long-lived and wide-ranging species is an essential component of effective population management. Open-source matching software coupled with matching algorithms exploiting the power of information theory will make this process more efficient and less prone to error. Our main objective was to provide a procedure for incorporating full matching uncertainty into the photo-identification process using a freely available and simple software package. Despite the relatively low number of photographs with which we tested our approach, the performance of the system is satisfactory from the perspective of estimating reliable demographic information for a host of wildlife species.

Our assessment of a simple, freely available spot pattern-matching software package coupled with an information-theoretic incorporation of matching uncertainty was particularly effective for whale sharks given that their natural spot patterns were ideally suited for assessment using the I^3^S program. Validation of I^3^S matches using the Information Criterion algorithm provided a threshold *w*_1 _for known matched pairs of approximately 0.2, below which *w*_1 _for non-matched pairs fell. Known matched pairs not matched by I^3^S, or that were matched with low (i.e., <0.2) *w*_1, _likely resulted from poor clarity or high angles of yaw. This emphasizes the need to select images of the highest quality for matching purposes [39]. The validation process is necessary with most computer-aided matching algorithms because this alleviates much of the subjectivity associated with the final stage of matching. In the case of whale sharks, the 0.2 threshold proved to be a robust and conservative measure of certainty, but the particular value of the threshold will likely vary among species. Nonetheless, in the absence of validation data we suggest that using this threshold value is a good first approximation.

The validation stage of photographic matching can be further confirmed by using genetic tagging to identify individuals [15], and this approach is proliferating in mark-recapture studies. Genetic tagging also has the advantage of providing additional individual- and population-level information (e.g., genetic diversity, parent-offspring relationships, etc.) [40]. Because whale sharks are highly photographed and tissue sampling may be difficult, it is unlikely that genetic tagging will replace photographic identification in the near future, even though genetic information will provide further validation of photographic matching success.

The open-source program I^3^S [32] was effective at confirming past matches made by eye in the majority of instances. Images that were successfully confirmed using our Information Criterion algorithm received relatively low *w*_1 _and *ER*_1 _overall, most likely as a result of a considerably smaller sample size than that used for validation. I^3^S was also a useful tool for identifying image matches that were assigned incorrectly (i.e., both false positives and false negatives). When matching whale shark patterns by eye, the observer generally does not focus on the spot pattern *per se*; rather, attention is usually paid to the intricate lines and whirls (see Fig. 1a) on the flank of the shark. As such, I^3^S provides an unbiased method of matching natural markings that is relatively immune to user subjectivity.

We found strong evidence that horizontal angle of subjects within images affects the ability of the I^3^S algorithm to make reliable matches. As the horizontal angle of subjects in images increases, the matching likelihood decreases. Angles of yaw up to 30° compromise the matching process even though many of these images were still matched correctly. Conversely, images with angles of yaw ≥40° will more than likely be incorrectly assigned. Due to the linear algorithm used by I^3^S to match spot patterns it is important to use only those photos with as little contortion of the reference area as possible. Likewise, the number of spots annotated in fingerprints can also potentially affect the I^3^S matching process. The higher the number of spot pairs matched, the lower the I^3^S score and hence, the higher the matching certainty. This corroborates similar findings from a study of *Carcharias taurus *[31] and emphasizes the benefit of using information-theoretic measures of matching parsimony because the updated algorithm takes relative match uncertainty into account.

The number of suitable images from our database for use in I^3^S was considerably reduced due to the absence of reference points, poor image quality and oblique angles of subjects in many images. The rejection rate is inflated particularly by the use of photographs taken without the explicit aim of photographic matching because many are derived from ecotourism operations. However, the efficiency and reliability of matching with I^3^S more than compensated for the reduced sample size. The number and size of images in an I^3^S database can potentially slow down the program's operating speed; therefore, it is ideal to scale down the size of photographs and only include the best image of a particular animal. In addition to horizontal angle, roll and pitch of sharks in images may affect the matching process. Pitch seems likely to be only a minor problem because digital photos can be rotated so that the animal is aligned with the horizontal. We had few images of the same individual at varying angles of roll, so we were unable to examine this potential problem.

## Conclusion

The application of I^3^S to any animal with a unique, stable spot pattern holds particular promise for mark-recapture studies. The program is particularly well suited to organisms that have minimal contortion in the desired reference area and have spots that are relatively homogenous in diameter and size. Large, irregular spots may cause problems during fingerprinting because the centre of the spot may vary according to the user's preference. For example, a species with a spot pattern that may not be well suited to I^3^S is the manta ray (*Manta birostris*) due to its large, sparsely spaced and irregular ventral spot patterns [41]. However, other species of ray such as the white spotted eagle ray (*Aetobatus narinari*) have evenly spaced and relatively homogenous spot patterns on the dorsal surface that would lend themselves more readily to the fingerprinting process. Other organisms that are potentially suitable candidates include: felids, some cetaceans, many birds, amphibians and reptiles, and other elasmobranchs.

The benefits of non-intrusive mark-recapture studies are numerous, not only in terms of animal welfare, but also from a logistical perspective. The software availability and applicability of I^3^S for a wide range of animals will enable researchers to store and match images for mark-recapture purposes, thus hopefully contributing to robust and more precise estimates of key life history parameters. Reliable, effective photo-identification for animals with stable, natural markings is now possible for anyone armed with a digital camera.

## Methods

### Whale shark photo library

The library contains 797 photos taken by researchers and tour operators during the months of March–July from 1992–2006 at Ningaloo Reef  (22º 50’ S, 113º 40’ E), Western Australia. The method of image capture varied over time, so that still, video and digital images were all included in the library. A 'by-eye' comparison of 581 images in this photo library, (this total excludes several images collected in the 2001 season, as well as all photos collected between 2003 and 2006), was originally completed. During analysis, photos were sorted into quality classes on the basis of clarity, angle, distinctiveness, partial image and overall quality [39]. More details of the manual matching procedure are provided in reference [19].

### Matching software and fingerprint creation

The software we used to generate potential image matches was originally designed to match natural variation in spot patterns of grey nurse sharks (*Carcharias taurus *– also known as the "ragged-tooth" in South Africa and the "sand tiger" shark in North America) [31]. This software – Interactive Individual Identification Software (I^3^S) – creates 'fingerprint' files and matches individuals by comparing particular areas demonstrating consistent spot patterns. We chose to examine the area on the flank directly behind the 5^th ^gill slit as the most appropriate for the individual identification of whale sharks. This decision was based on spot consistency identified in previous studies and due to the ease with which photographers can view this area [19,20]. The positioning of spots in this area was also less likely to be distorted due to undulation of the caudal fin, which may affect the software's matching success.

At least three reference points are required by I^3^S to construct a fingerprint [31]; we chose the most easily identifiable and consistent reference points visible in flank photographs: 1) the top of the 5^th ^gill slit, 2) the point on the flank corresponding to the posterior point of the pectoral fin and 3) the bottom of the 5^th ^gill slit (Fig. 1a). The requirement of all three reference points to be visible in the photograph for a fingerprint to be created meant that not all 797 photos could be used. As such, we could compare 433 (54%) of the original photographs, of which 212 were of the left side (LS) and 221 were of the right side (RS) of the shark.

In this updated database, images were matched by an operator highlighting spots within the reference area on a computer screen. Three initial reference points for each image were entered (Fig. 1a), followed by the manual adding of a digital point to the centre of the most obvious spots within the reference frame. Using a search function, the software compares the new fingerprint file against all other fingerprint files in the database by using a two-dimensional linear algorithm, which is simply the sum of the distances between spot pairs divided by the square of the number of spot pairs [31]. The matched spot pairs with the minimum overall score (ranging from 0 [perfect match] to a value <1) is the most likely match. The program also lists the next 49 most likely image matches, which it ranks in decreasing order of likelihood. A search result output text file provides a list of the 50 matches, spot pairs compared, as well as a matching score. We then incorporated the I^3^S text output into the *R *Package [42] for further analysis [see Additional file 1].

### Information criterion algorithm

To provide a measure of match parsimony based on the philosophy of information theory and to compare possible image matches in a multi-model inferential framework [27], we modified the match score in the following manner: (1) we first back-transformed the spot-averaged sum of distances to a residual sum of distances, which was simply the spot score (*SS*) multiplied by the square of the number of matching spots (*n*); (2) we then created an information criterion (IC) analogous to the Akaike and Bayesian Information Criteria [43,44]:

IC=2k+n′loge(SS⋅n2n′)
 MathType@MTEF@5@5@+=feaafiart1ev1aaatCvAUfKttLearuWrP9MDH5MBPbIqV92AaeXatLxBI9gBaebbnrfifHhDYfgasaacH8akY=wiFfYdH8Gipec8Eeeu0xXdbba9frFj0=OqFfea0dXdd9vqai=hGuQ8kuc9pgc9s8qqaq=dirpe0xb9q8qiLsFr0=vr0=vr0dc8meaabaqaciaacaGaaeqabaqabeGadaaakeaacqqGjbqscqqGdbWqcqGH9aqpcqaIYaGmcqWGRbWAcqGHRaWkcuWGUbGBgaqbaGqabiab=XgaSjab=9gaVjab=DgaNnaaBaaaleaacqWGLbqzaeqaaOWaaeWaaeaadaWcaaqaaiabdofatjabdofatjabgwSixlabd6gaUnaaCaaaleqabaGaeGOmaidaaaGcbaGafmOBa4MbauaaaaaacaGLOaGaayzkaaaaaa@4462@

where *k *= an assumed number of parameters under a simple linear model (set to 1 for all models) and *n*' = 100/*n *that accounts for the fact that an increasing number of spots automatically leads to a higher *SS *(the 100 multiplier scales the term to be >1); (3) finally, we calculated the IC weight (*w*) as:

wi=e−0.5⋅ΔICi∑i=1mΔICi
 MathType@MTEF@5@5@+=feaafiart1ev1aaatCvAUfKttLearuWrP9MDH5MBPbIqV92AaeXatLxBI9gBaebbnrfifHhDYfgasaacH8akY=wiFfYdH8Gipec8Eeeu0xXdbba9frFj0=OqFfea0dXdd9vqai=hGuQ8kuc9pgc9s8qqaq=dirpe0xb9q8qiLsFr0=vr0=vr0dc8meaabaqaciaacaGaaeqabaqabeGadaaakeaacqWG3bWDdaWgaaWcbaGaemyAaKgabeaakiabg2da9maalaaabaGaemyzau2aaWbaaSqabeaacqGHsislcqaIWaamcqGGUaGlcqaI1aqncqGHflY1cqqHuoarcqqGjbqscqqGdbWqdaWgaaadbaGaemyAaKgabeaaaaaakeaadaaeWbqaaiabfs5aejabbMeajjabboeadnaaBaaaleaacqWGPbqAaeqaaaqaaiabdMgaPjabg2da9iabigdaXaqaaiabd2gaTbqdcqGHris5aaaaaaa@4967@

where ΔIC = IC - IC_*min *_for the *i*^th ^image (*i*^th ^'model') from 1 through *m *(where *m *= 49). We also calculated the information-theoretic evidence ratio (*ER*) [27] for each matched image relative to the top-ranked image based on the *w *to provide a likelihood ratio of match performance. Here, *ER*_1 _is the *w *of the top-ranked matched photograph divided by the next most highly ranked photograph's *w*, *ER*_2 _is the *w *of the top-ranked match divided by the *w *of the third-best match, and so on. Therefore, *ER*_1 _provides a likelihood ratio for the match of the top-ranked photograph relative to the next most highly ranked photograph.

### Match validation

To establish the ability of the *w*_*i *_and *ER *indices to assign reliable matching, we endeavoured to establish a threshold value of *w*_1 _and *ER*_1 _below which matching uncertainty was too high to match photographs reliably. We therefore validated the approach by applying our algorithms to a sample of 200 images; 25 known matched pairs (i.e., matched by eye) from both the LS and RS databases (100 images total), and 25 non-matched pairs from both LS and RS databases (100 images total). The LS and RS images were analyzed separately, using text outputs from I^3^S that report the candidate matching image names, I^3^S matching scores and the number of spot pairs matched. A match was considered successful if the corresponding image was ranked at the top of the list of potential matches (i.e., number 1 of 50).

### Assessing 'by-eye' matches using I^3^S

Thirty-three individual sharks were re-sighted inter-annually during the manual 'by-eye' analysis of the raw photo library. Of any two by-eye matched images, one of the pair was entered into either the LS or RS database and searched. A match using I^3^S was considered successful if the by-eye matched images were ranked as the most likely match (as with the validation test) and confirmed using the IC algorithm.

### Horizontal angle (yaw)

Footage of 10 different sharks (5 LS and 5 RS) was used to capture sequences of five images of each shark, where subjects were on varying horizontal angles (0°, 10°, 20°, 30° and 40° – Fig. 2). The angles of yaw were estimated using Screen Protractor™ software. Fingerprints were created for each image with 20 spots annotated per fingerprint. The 10° images were searched against the 0° images and 10 non-matching images. This process was repeated, substituting images where subjects were on angles of 20°, 30° and 40° for both LS and RS image sequences. Five random, non-matching pairs were also searched against 0° and 10° images, and then repeated for 20°, 30°, and 40° images. This allowed for a comparison between matching and non-matching pairs while testing for the effects of horizontal angle in images. Results were analyzed using the IC algorithm applied to the match validation and by-eye comparison tests.

### Number of spot pairs

Fifty known-matching pairs were compared to one another in I^3^S. Of these matching pairs, only those successfully confirmed during validation of I^3^S matches were included in this test. I^3^S scores were compared against the number of spot pairs matched. The *w*_1 _for each image was also compared against the number of spot pairs matched by the I^3^S algorithm. A complementary log-log transformation (clog-log) was applied to normalize the distribution of I^3^S scores and *w*_1_, and a log_10 _transformation was used to normalize the distribution of spot pairs. We tested for a linear relationship between the transformed variables using least-squares regression and information-theoretic evidence ratios. Goodness-of-fit was assessed using the least-squares R^2 ^value.

## Competing interests

The author(s) declare that they have no competing interests.

## Authors' contributions

CWS, CJAB and MGM designed the study, CWS and CJAB did the analysis, and all authors contributed to writing the paper. CWS did most of the analysis with assistance from CJAB, and CWS took the lead in writing the manuscript.

## Supplementary Material

Additional file 1 ***R *code to calculate Information Criterion (IC) weights for match parsimony**. Full instructions for use of *R *code are contained within the text file. Click here for file
